# Surface Morphology of Textured Transparent Conductive Oxide Thin Film Seen by Various Probes: Visible Light, X-rays, Electron Scattering and Contact Probe

**DOI:** 10.3390/ma15144814

**Published:** 2022-07-10

**Authors:** Krunoslav Juraić, Pavo Dubček, Mario Bohač, Andreja Gajović, Sigrid Bernstorff, Miran Čeh, Aden Hodzic, Davor Gracin

**Affiliations:** 1 Ruđer Bošković Institute, Bijenička Cesta 54, 10000 Zagreb, Croatia; pavo.dubcek@irb.hr (P.D.); mario.bohac@irb.hr (M.B.); andreja.gajovic@irb.hr (A.G.); davor.gracin@irb.hr (D.G.); 2 Elettra-Sincrotrone Trieste, SS 14, km 163.5, 34149 Trieste, Italy; sigrid.bernstorff@elettra.eu; 3 Jožef Stefan Institute, Jamova 11, 1000 Ljubljana, Slovenia; miran.ceh@ijs.si; 4 Central European Research Infrastructure Consortium (CERIC-ERIC), 34149 Trieste, Italy; aden.hodzic@ceric-eric.eu

**Keywords:** surface morphology, surface texture, thin films, tin oxide, roughness, TEM, GISAXS, AFM, UV-Vis-NIR light scattering, fractal dimension, haze ratio

## Abstract

Fluorine-doped tin oxide thin films (SnO_2_:F) are widely used as transparent conductive oxide electrodes in thin-film solar cells because of their appropriate electrical and optical properties. The surface morphology of these films influences their optical properties and therefore plays an important role in the overall efficiencies of the solar cells in which they are implemented. At rough surfaces light is diffusely scattered, extending the optical path of light inside the active layer of the solar cell, which in term improves light absorption and solar cell conversion efficiency. In this work, we investigated the surface morphology of undoped and doped SnO_2_ thin films and their influence on the optical properties of the films. We have compared and analysed the results obtained by several complementary methods for thin-film surface morphology investigation: atomic force microscopy (AFM), transmission electron microscopy (TEM), and grazing-incidence small-angle X-ray scattering (GISAXS). Based on the AFM and TEM results we propose a theoretical model that reproduces well the GISAXS scattering patterns.

## 1. Introduction

Transparent conductive oxides (TCO) are a group of metal oxides (In_2_O_3_, ZnO, SnO_2_, CdO, GaInO_3_, CdSb_2_O_3_, etc.) that have appropriate optical and electrical properties [1,2]. They have wide optical band gaps and are transparent in the visible part of the electromagnetic spectrum, and it is common practice to influence their electrical conductivity by doping with metal(Sb, Nb, Sr, W, Ta, La, Li, Ga) and non-metal atoms (F, P, Mo, Co, etc.) [3]. In the form of thin films, they can be prepared by various chemical and physical methods. The most commonly used are: magnetron sputtering, spray pyrolysis, sol-gel, thermal or electron-beam evaporation, pulsed laser deposition, hydrothermal processing, spin coating, drop coating, deep coating, screen printing, atomic layer deposition [1].

TCO thin films have been intensively investigated for many years and have many applications in everyday life, due to the optimal balance between electrical conductivity and optical transparency. They are widely used in opto-electrical devices such as gas sensors, flat panel displays, front electrodes in solar cells, catalyst supports, etc. [4,5].

The most widely used TCO is indium tin oxide (ITO), but due to the increasing price of indium and its pure chemical resistance and thermal stability [6], other TCOs have emerged as suitable alternatives, such as aluminium-doped zinc oxide (AZO) and fluorine-doped tin oxide (SnO_2_:F). SnO_2_:F is chemically inert, scratch-resistant, and can withstand high temperatures [5]. It has a wide optical bandgap (about 3.7 eV) and is transparent in the whole visible range [7]. SnO_2_:F is often used as a TCO front electrode in thin-film solar cells (a-Si:H) [8] and as an electron transport layer (ETL) in new generation solar cells: perovskite solar cells [9,10], dye-sensitised solar cells [11] and organic solar cells [12].

In addition to transparency in the visible range (>80%), its rough surface enhances diffuse light scattering, which extends the path of the light within the active part of the solar cell and thus increases the conversion efficiency of the solar cell. The intensity of the diffusely transmitted light is strongly dependent on the surface morphology. The angular distribution of light in the UV-Vis-NIR part of the spectrum scattered (transmitted and reflected) by thin films on a thick substrate depends strongly on the top surface and the other interface morphology. The intensity of diffusely scattered light can be enhanced by rough surfaces of thin films through reduced reflection and enhanced scattering [13,14]. Therefore, it is crucial to have a detailed insight into the surface morphology of TCO thin films for application in optoelectronic devices.

In this work we applied three different techniques: transmission electron microscopy (TEM), atomic force microscopy (AFM), and grazing incidence small-angle X-ray scattering (GISAXS) [15,16] to analyse the surface morphology of SnO_2_ thin films deposited on soda-lime glass substrates by atmospheric pressure chemical vapour deposition (APCVD). By varying the deposition parameters during APCVD, we prepared a series of SnO_2_ thin films with varied surface morphologies (roughness, grain shape and size, thickness).

In our previous publication [17], we analysed and discussed the magnetotransport properties (charge carrier density and mobility extracted from DC resistivity and Hall effect measurements) of SnO_2_ thin film samples (undoped single-layer and doped/undoped bi-layer samples) and correlated them to the crystal structure (GIXRD) and chemical content (TOF-ERDA). The results showed that undoped single-layer samples are not suitable for application as TCO electrodes in solar cells. Therefore, an additional doped layer is added. TOF-ERDA results showed that the samples have the stoichiometric ratio of Sn and O atoms (1:2, within the measured error, taking into account that some of the O atoms are bonded to Si in the substrate or to C atoms on the sample surface). In the samples deposited in the two-step process, the concentration of F atoms (dopant) is almost 1%, while in the samples deposited in the one-step process, the amount of F atoms is below the detection limit (<0.1 at.%).

In this work, the focus is on the surface morphology and its influence on the optical properties (diffuse light scattering) measured by UV-Vis-NIR direct and diffuse light transmittance. We have compared the results of the surface morphology analysis obtained by all of the mentioned methods (TEM, AFM, GISAXS) and explained the discrepancies, highlighting the important characteristics (advantages and disadvantages) of the experimental techniques used. Based on the results of TEM, AFM, and GISAXS 1D cross-section analysis, we proposed a unique theoretical model that reproduces the GISAXS 2D patterns very well. The transmittance data are modelled by applying a simple theoretical model that takes into account the influence of surface roughness.

## 2. Materials and Methods

### 2.1. Sample Preparation

All samples were deposited by an industrial (large-scale) APCVD system, which is widely used because it does not require a vacuum, its low cost, and its overall simplicity. The reacting gas mixture (SnCl_4_, H_2_O, methanol, and oxygen) was produced in a bubbler and sprayed through APCVD reactor nozzles on soda-lime glass substrate heated at 590 °C and 610 °C in the APCVD reactor oven. By varying the deposition parameters (substrate temperature and spraying mixture content), we obtained coatings with different surface textures (Table 1). Samples S-590 and S-610 are made in a single-step process as an undoped single layer. The other two samples (B-590 and B-610) have an additional doped layer deposited on top (Table 1). Fluor was added to the reacting mixture during the doped top layer deposition. More details about the deposition procedure can be found in ref. [17].

Prior to the deposition, the soda-lime glass substrates were cleaned in a standard way (three-step protocol). In the first step, the glass substrates were washed with a detergent solution, in the second step with isopropanol, and in the third step with milli-Q water.

### 2.2. Transmission Electron Microscopy and Selected Area Electron Diffraction (TEM/SEAD)

For TEM observation, all samples were prepared in cross-section geometry. Two parts of the same thin-film sample were glued together, cut by ultrasound drill, and embedded into copper rings using epoxy. The samples were then thinned to a thickness of 70 µm and dimpled using a Gatan dimpler to a thickness of app. 20 µm. Ion-milling was used to perforate the samples to electron transparency. The ion-milling conditions were: 4 kV followed by 1 kV, incident angle of ions 10 degrees, and current 1 mA.

TEM and selected area electron diffraction (SAED) measurements were made using Jeol JEM-2100 and Jeol 2010F transmission electron microscopes (Jeol Ltd., Tokyo, Japan) at 200 kV under the same conditions and magnifications to allow comparison of the results. Bright-field, as well as dark-field imaging techniques, were used.

### 2.3. Atomic Force Microscopy (AFM)

Atomic Force Microscopy was done using a Nanoscope IIIa controller (Veeco Instruments, Santa Barbara, CA, USA), silicon-nitride tip (NP-20, Veeco, k = 0.32 N/m) in the contact mode. The size of the scanned area was 10 × 10 μm (512 × 512 pixels). The AFM image analysis was done using Gwyddion 2.60 software (P. Klapetek, Czech Metrology Institute, Brno, Czech Republic and D. Nečas, University of Technology, Brno, Czech Republic) [18]. Before quantitative analysis, AFM images were corrected for polynomial background (second-order) to avoid the possible influence of the surface curvature. Also, the data were levelled by subtraction of the mean plane.

### 2.4. Grazing Incidence Small Angle X-ray Scattering (GISAXS)

The thin film samples were thoroughly examined by grazing incidence small angle X-ray scattering (GISAXS). The GISAXS patterns were obtained with synchrotron radiation at the Austrian Small Angle X-ray Scattering (SAXS) beamline at the Synchrotron Elettra (Trieste) using 8 keV X-rays [19]. The q-scale was calibrated by taking SAXS diffraction patterns of rat tail tendon fibre collagen. The distance between the sample and the detector was 1750 mm. The beam size was 4 mm × 150 µm. The GISAXS patterns were recorded with a CCD detector (1024 × 1024 pixels, 12-bit dynamic range, 4096 counts per pixel). The final image was calculated as an average of 64 subsequently acquired frames to enhance the signal-to-noise ratio eight times. The angle of grazing incidence was varied from the critical angle for total external reflection α_C_ to α_C_ + 0.3° to reach also the part of the samples below the surface. At the critical angle, the photon beam penetrates only 10–20 nm below the surface and thus provides information about the surface morphology. For the maximum angle of grazing incidence (α_C_ + 0.3°), the beam enters into the film where it is exponentially depleted, with a nominal penetration depth of more than 100 nm, depending on the morphology of the surface itself. Therefore, the GISAXS pattern contains the morphological information for the whole SnO_2_ layer down to the film-substrate interface. See ref. [20] for more details about the experimental setup.

### 2.5. UV-Vis-NIR Transmittance

The optical properties and thickness of the SnO_2_ thin films were determined from measurements of the specular and total transmittance (specular + diffuse) in the UV-Vis-NIR part of the spectrum (340–950 nm). The total transmittance was measured with an integrating sphere (diameter 80 mm) connected to a UV-Vis-NIR spectrometer (Ocean Optics HR-4000, Ocean Optics, Inc., Dunedin, FL, USA) and using a tungsten/halogen light source (Ocean Optics HL-2000, Ocean Optics, Inc., Dunedin, FL, USA) with optical fibres. The thickness of the SnO_2_ layers was estimated from the position of the interference minima and maxima in the transmittance spectra using the envelope method [21].

## 3. Results and Discussion

### 3.1. TEM/SAED

**Figure 1:**
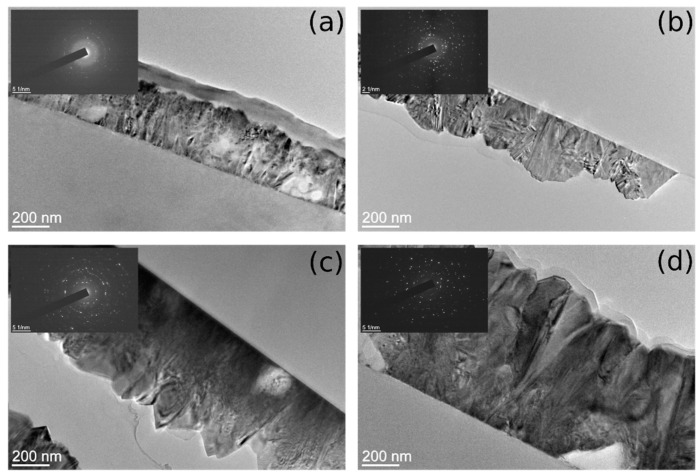
Cross-section TEM images of SnO_2_ samples: (**a**) S-610 (**b**) S-590, (**c**) B-610 and (**d**) B-590. Corresponding SEAD patterns are shown in the insets. The average thickness and RMS roughness (calculated as standard deviation of the surface border distance from the SnO_2_ layer—glass interface) calculated from the TEM images are: (**a**) (308 ± 18) nm, (**b**) (270 ± 40) nm, (**c**) (582 ± 51) nm and (**d**) (811 ± 36) nm.

Cross-section TEM images of the prepared SnO_2_ samples are presented in Figure 1. The samples vary in film thickness as well as in grain size. The sample thickness, estimated qualitatively from the TEM images for all investigated samples, ranged between 270 nm and 811 nm (Table 1). The films are polycrystalline in nature, which is resolved from the corresponding electron diffractogram (SAED) presented as insets for each sample subfigure. Grain sizes are smaller near the substrate as compared with the grain sizes near the surface of the film. All films exhibited rough surfaces (in the range 18–51 nm) as a consequence of the faceting of the surface grains.

Single-layer samples (Figure 1a,b) have a rough surface with more rounded shapes (random) on the surface compared to double-layer samples (Figure 1c,d) that display sharp peaks with flat surfaces related to crystallites corners. SEAD patterns (inset of TEM images in Figure 1) confirm that the samples consist of SnO_2_ crystallites that are randomly oriented. The intensity and the number of reflection (diffraction) spots are higher for double-layer samples indicating a larger number of crystallites.

**Figure 2:**
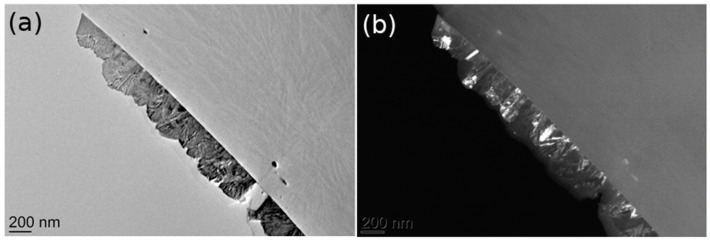
TEM cross-section images of a CVD film. (**a**) bright-field image and (**b**) the corresponding dark-field image.

The crystallographic relations between individual grains were studied using the dark-field imaging technique (Figure 2). Typical pairs of bright-field and dark-field TEM images of the CVD films are shown in Figure 2. The grains that are in the zone axis exhibit high intensity in the dark-field image. Columnar-shaped grains within the film are, in fact, composed of different crystallographically oriented grains and do not exceed 100 nm in size.

The surface of the double-layer samples consists of well-defined inclined surfaces (up to 200 nm in size) forming large pyramids, while for the single-layer samples, that is not the case. For the single-layer samples, the large sharp pyramidal shapes are not so pronounced. Pyramidal shapes correspond to corners of crystallites that are grown up from the film surface. The single-layer samples do not consist of so many nanocrystals as it is indicated by SEAD and the sharp pyramidal shapes do not dominate on the surface. Single-layer samples are thinner and correspond to the early (starting) stage of crystallite growth when there are fewer crystallites with a smaller average size, while double-layer samples are much thicker and correspond to a later stage of crystallites growth when the crystallites are well-formed.

### 3.2. Atomic Force Microscopy

**Figure 3:**
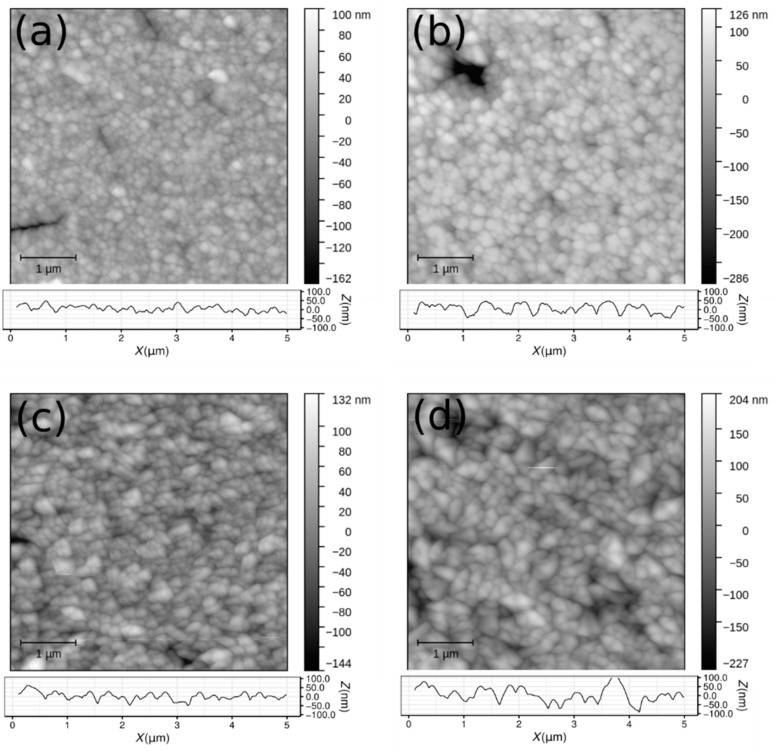
D AFM images of SnO_2_ thin film samples surface: (**a**) S-610, (**b**) S-590, (**c**) B-610, (**d**) B-590. 1D horizontal profile is added below each 2D image.

The surface morphology of the SnO_2_ samples was investigated by AFM (Figure 3). The surface is uniform and homogeneous with some isolated defects (deeper holes or spikes) as can be seen in Figure 3b (upper left corner).

The surface morphology was quantitatively analysed by the calculation of well-known roughness parameters: average roughness (*σ_a_*) and root mean square (RMS) roughness (*σ_q_*). *σ_a_* is defined as an average height deviation from the mean plane and *σ_q_* as standard deviation of the height distribution:

(1) $$\sigma_{a}=\frac{1}{A}\iint_{A}^{}\left|z\left(x,y\right)\right|\partial x\partial y=\frac{1}{NM}\overset{N}{\underset{i=1}{\sum }}\overset{M}{\underset{j=1}{\sum }}\left|z_{i,j}\right|$$

(2) $$\sigma_{q}=\sqrt{\frac{1}{A}\iint_{A}^{}{\left(z\left(x,y\right)\right)}^{2}\partial x\partial y}=\sqrt{\frac{1}{NM}\overset{N}{\underset{i=1}{\sum }}\overset{M}{\underset{j=1}{\sum }}{\left(z_{i,j}\right)}^{2}}$$

where *x* and *y* are in-plate coordinates and *z* is the height. The integration is over the sample surface, *A*, but in practice, it is calculated as a discrete sum over all nodes in the AFM image. All parameters were calculated as an average value obtained by analysis of three AFM images taken at different positions on the sample surface. The obtained surface morphology parameters (surface roughness, effective surface area) are summarised in Table 2.

The average surface roughness *σ_a_* (Equation (1)) for all analysed samples is in the range (13.7–34.2) nm and is systematically lower than the RMS surface roughness *σ_q_* (Equation (2)) which is in the range (19.3–43.9) nm. Higher deposition temperature produces samples with lower surface roughness, but this can be also explained by the influence of the lower thickness of these samples and the properties of columnar growth of the SnO_2_ layer. Significantly larger values of *Z_max_ − Z_min_* compared to the surface roughness correspond to rare deeper holes and higher spikes in AFM images (Figure 3).

Besides the surface roughness (a parameter that describes the surface morphology and which is scale-dependent), we have calculated the fractal dimension for the AFM images. The fractal dimension is a parameter that characterises fractal patterns or sets by quantifying their complexity as a ratio of the change in detail to the change in scale [22]. For two-dimensional objects like surfaces, the fractal dimension has a value between 2 and 3 and provides information about the irregularity of the surface morphology. A larger value indicates a more irregular and fragmented surface. For example, a surface with a fractal dimension close to 2 fills space very much like an ordinary surface, but if a surface has a fractal dimension close to 3 folds and flows to fill space rather nearly like a volume [23]. There are several methods for fractal dimension calculation: the cube counting method [24], the triangulation method [24], variance method [25], power spectrum [25], the autocorrelation method [26], the structure factor method [27], and roughness scaling method [28]. Some of them we have applied to SnO_2_ layers.

**Figure 4:**
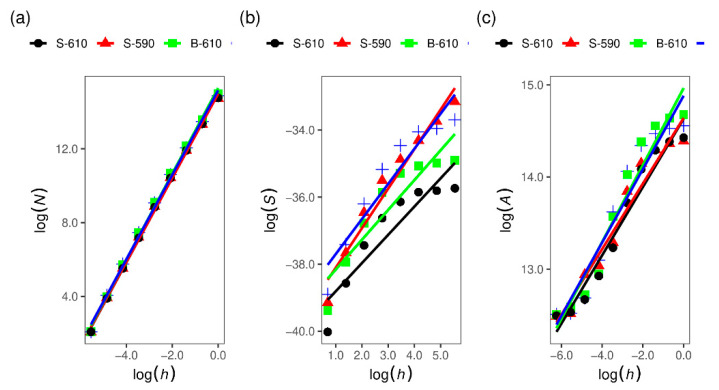
Fractal dimension calculation by: (**a**) cube counting method, (**b**) variance method and (**c**) triangulation method. Obtained values for fractal dimensions are summarised in Table 2.

Results of the fractal dimensions calculation (Figure 4) using methods implemented in the Gwyddion software are summarised in Table 2. The fractal dimensions for all samples are between 2 and 3 determined by all applied calculation algorithms and such values are expected for thin film with rough surfaces. Systematic differences between the calculation algorithms are expected. The cube counting method provides the smallest values for the fractal dimension, while the variance method provides the largest values. In addition, the sample with the smallest thickness, surface roughness, and z range has also the smallest value of the fractal dimension. Samples deposited at a lower temperature (both single- and double-layer samples) have a higher surface roughness and also fractal dimension.

### 3.3. Grazing Incidence Small Angle X-ray Scattering

**Figure 5:**
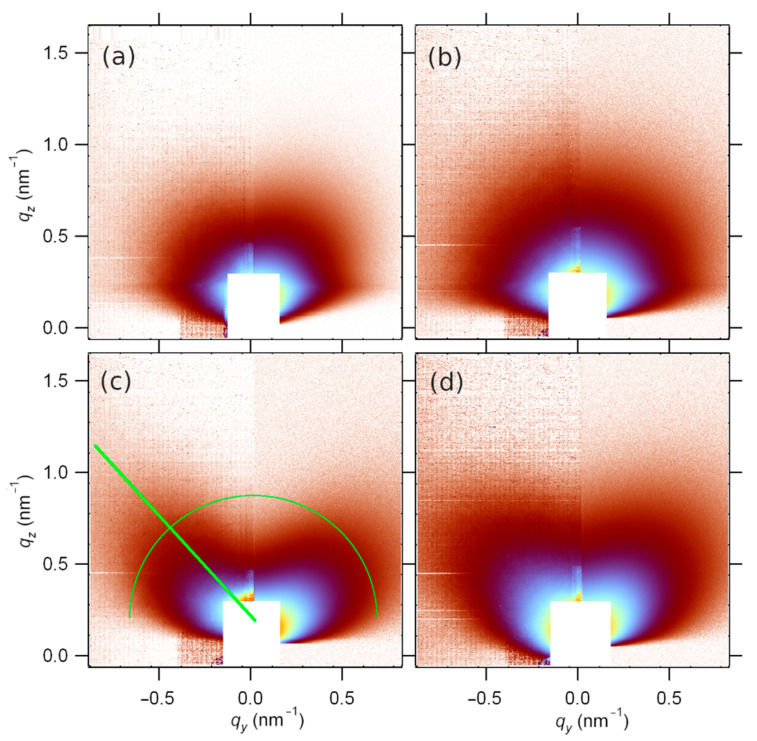
GISAXS pattern for SnO2 single- and double-layer films on glass substrate for a grazing angle of incidence equal to αc + 0.1°: (**a**) S-610, (**b**) S-590, (**c**) B-610, (**d**) B-590. The left side of each subfigure represents experimentally obtained data, and the right side represents the result of the theoretical model application. The green line and semicircle indicate the positions where the scattered intensity variation is extracted and presented in Figure 6 and Figure 7. The white rectangle at the q space origin marks the detector area covered by an Al beamstop in order to protect the detector from a very strong directly reflected X-ray beam.

In addition to the before-mentioned detailed surface morphology analysis by TEM and AFM and structural analysis by SAED and dark field TEM, some additional information about the thin-film morphology and the surface distribution of crystallites can be obtained from GISAXS. The off-specular GISAXS scattering is caused by the surface morphology (surface roughness and shapes present at the surface). The results of the GISAXS experiment are shown in Figure 5, where the left side of each image is the measured scattering, while the right side is the model scattering discussed later. The single-layer SnO_2_ thin films have the scattered intensity evenly distributed in all directions (Figure 5, samples S-610 and S-590) suggesting either spherical or randomly oriented crystallites in the surface layer.

Regarding the angle of grazing incidence, there is no significant difference and deviation from patterns presented in Figure 5, between GISAXS patterns taken for the smallest angle of grazing incidence equal to the critical angle for total external reflection and for the highest applied angle. In fact, because of the very high roughness and large inclined flat areas belonging to the pyramid shapes at the surface, the surface level is not well defined and, accordingly, the angle of grazing incidence varies from position to position at the pyramidal grains. The incident X-ray beam is mainly transmitted through inclined grain surfaces, reducing the influence of the angle of grazing incidence on the GISAXS pattern.

**Figure 6:**
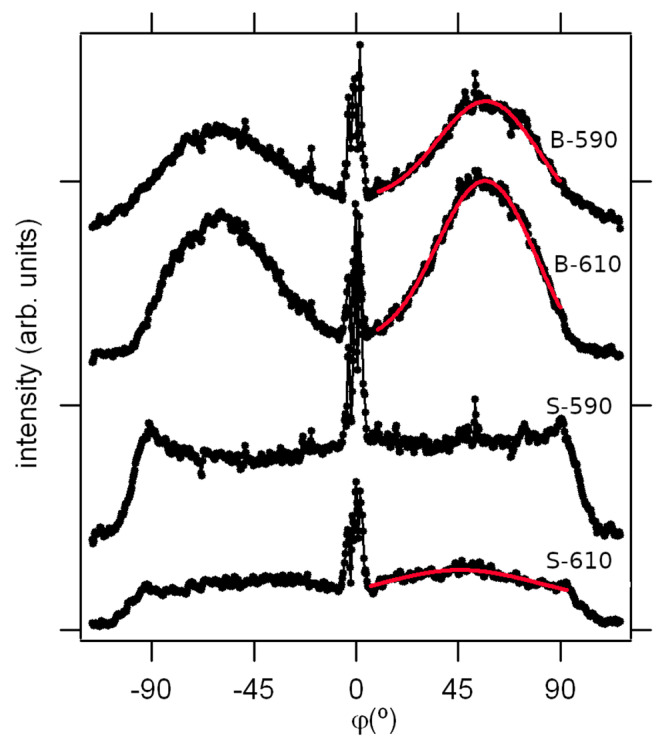
All angle (circular) section (*q_y_*^2^ + *q_z_*^2^ = const.), taken over semi-circular path marked in Figure 5, indicating the preferred orientation. Red lines are the Gaussian fits.

There is a weak indication of some preferred scattering direction for S-590 in the radial direction at about 45° inclined to the specular plain. Contrary to that, the double-layer films show totally different GISAXS patterns (Figure 5c,d). The scattered intensity has a maximum value in the direction at the roll angle of about 55° with respect to the specular plain, indicating the preferred orientation of crystallites on the sample surface. This can be better seen in Figure 6 where the intensities along the green circle shown in Figure 5 are displayed for all the samples as a function of roll angle. This anisotropy is more pronounced for the sample deposited at a higher temperature (B-610).

Supposing a random deviation from the preferred orientation, the roll angle intensity distribution is fitted to a Gaussian superimposed on a constant baseline (Figure 6). For the S-610 sample, the fit is centred at 45°, and is 49° wide, as opposed to the double-layer samples B-610 and B-590, where the fits are centred at 56° and are 28° wide, with virtually equal Gaussians, the only difference is in the relative contribution of the baseline.

The signal in and near the specular plane (*q_y_* = 0) is considerably weaker indicating that the surface is not flat as it is for standard thin-film samples or glass substrates. Instead, it consists of relatively large fractions of flat but inclined areas as opposed to randomly rough surfaces.

An additional difference between single- and double-layer samples is concerning the Yoneda maximum [29] at *q_z_* ≈ 0.4 nm^−1^, where the scattering is enhanced at the critical angle. It is visible for the single-layer samples because of the smaller value of RMS roughness, while it vanishes for the double-layer samples.

**Figure 7:**
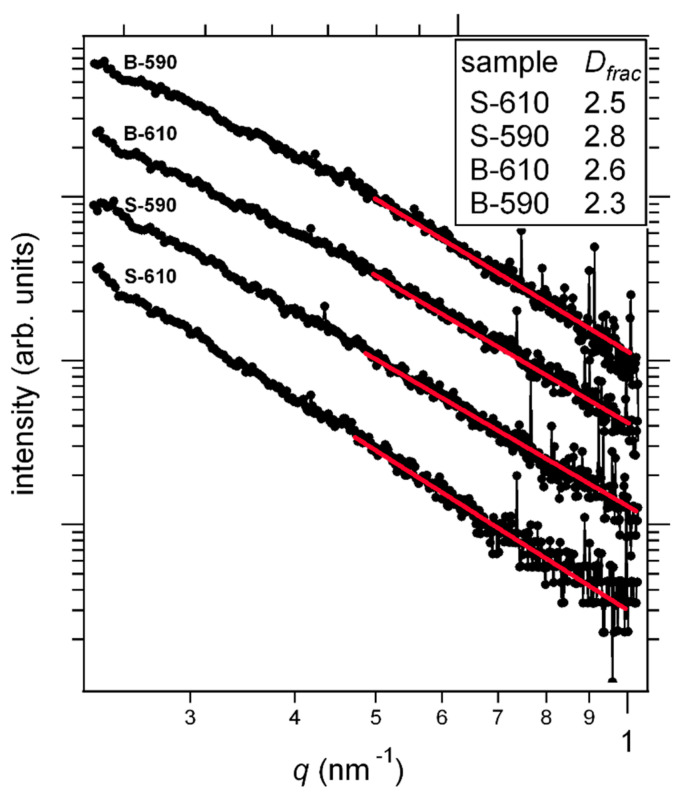
Porod plot for 1D intensity cut in the direction of the green line in Figure 5. The intensities have been offset vertically for clarity. Red lines represent the best fit to Equation (3).

Further GISAXS analysis was done by taking a 1D section along the direction of the maximum scattering, which is also indicated in Figure 5 by the green line and is 56° inclined to the specular plain. The resulting intensities are shown in Figure 7 on a log-log scale. The linearity of the plot means that we are looking at the so-called Porod tail where *q*·R > 1 and the scattered intensity decays by a power law (*q*^−4^ for smooth surface objects) regardless of the size and the shape of the scattering objects [30]. The information about the sizes is to be sought at smaller angles. Since the fits reveal a deviation from the *q*^−4^ dependency, we can tell something about the objects’ surface. Given the exponent values, we can conclude that the surfaces are sharp (there is an abrupt transition in the density at the surface), but they are not smooth, as they have some surface fractal shapes. The dimension of the fractals can be deduced from the fit [31]:

(3) $$I\sim q^{-P}=q^{D-6}$$

where *I* is the scattering intensity, *q* is the scattering wavevector. *P* is the Porod exponent and *D* is the fractal dimension. Evidently, all the features of the surface are considerably bigger than 10 nm, which is the limit imposed by the critical angle refraction: due to the grazing incidence, the scattering angles are practically limited to values above the critical angle. Therefore, also suggested by TEM results, we model our surfaces as a mixture of two contributions: 1. randomly oriented (including inclination) irregular pyramids and 2. regions of relatively large flat areas inclined to the surface normal (or specular plain), that is, big pyramids placed mostly vertically. Due to the pyramid sizes, the absorption cannot be neglected, and it is a function of the scattering angle.

In order to include absorption effects into the GISAXS intensity calculation for large pyramids on a flat surface, one has to divide the model sample into multiple layers which contain the slabs of the sliced pyramids. Then all the slabs scatterings from top to bottom are integrated, taking into account the variation of the effective layer density [32]. When the pyramids are big and randomly oriented, the scattering itself will be an isotropic contribution of the Porod tail type, and the Yoneda maximum will be smeared according to the slow density variation as a function of the depth.

On the other hand, for big vertical pyramids, the scattering contribution can be reduced to the Porod tail intensity distribution in the direction perpendicular to the sides of the pyramids. Given the considerable Gaussian widths obtained from Figure 6, this variation is ascribed to the roll angle distribution of the orientations of the scattering objects. These can be visualised as large hills in the shape of irregular and truncated pyramids. When hill-to-hill distances are larger than the coherence width, only absorption effects come into play. Again, we can slice the pyramids into horizontal slabs and integrate the absorption of the scattered intensity from a certain depth, for the given size distribution of hills. There will be no Yoneda maximum contribution.

The results of the model are shown in Figure 5 on the right side of each GISAXS image. Here, the intensity is a mere sum of intensities scattered from vertically (*I_Ver_*) and randomly (*I_Iso_*) oriented pyramids contribution:

(4) $$I=\eta I_{Ver}+\left(1-\eta \right)I_{Iso}$$

Double layered samples (B-590 and B-610) can successfully be modelled by uncorrelated pyramids since the Yoneda maximum is absent. The vertical pyramids were allowed to randomly deviate 30° from vertical orientation according to Figure 6 for both B-610 and B-590. The difference was only in the relative contribution of the oriented pyramids.

Single-layer samples (S-610 and S-590) scattering models are those of poorly correlated, randomly oriented slopes, and only S-610 has a minor contribution of preferred orientation, but with considerably a wide tilting range as obtained from the fit in Figure 5.

### 3.4. Optical Properties

**Figure 8:**
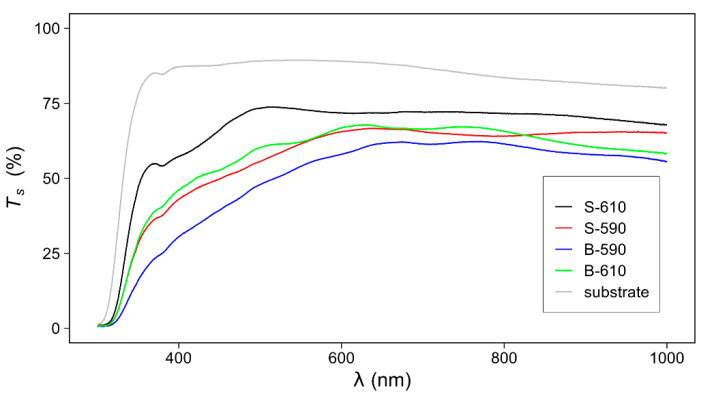
Measured transmittance *T_s_* in the UV-Vis-NIR spectral range for SnO_2_ thin film samples. The transmittance of the glass substrate is added for comparison.

The optical properties of the SnO_2_ (transmittance) samples are highly influenced by the surface morphology (mainly surface roughness). Figure 8 represents the obtained optical transmittance T_s_(λ) as a function of wavelength for the TCO thin-film layers on glass substrate.

The sharp decrease of the sample transmittance (SnO_2_ film + glass substrate) at lower wavelengths (<350 nm) is related to the existence of the optical band gap. Additional influence on the transmittance T_s_ in the UV range has the surface roughness, which leads to diffuse light scattering. The decrease in transmittance is most pronounced for the sample with the highest surface roughness and least pronounced for the sample with the lowest surface roughness. The decrease in transmittance for higher wavelengths (>700 nm) is due to absorption in the 3-mm thick glass substrate (soda-lime glass). In this range, the influence of surface roughness is not so pronounced and the variations of T_s_ are mainly related to the different thicknesses of the samples. Samples prepared at higher temperatures (S-610 and B-610) have a higher transmittance compared to samples deposited at lower temperatures (S-590 and B-590), which is related to the smaller thickness of the samples deposited at higher temperatures. The minima and maxima of the interference fringes, in general, are less pronounced than for a sample with a completely flat surface, especially for the sample with the highest surface roughness (Table 3, obtained by AFM).

Table 3 summarises the results of the quantitative analysis of the UV-Vis-NIR experiment. The optical band gap (*E_g_*) is calculated using the Tauc relation [33] and the index of refraction using the reflectance data in the weak absorption region [34]. For all samples, the optical band gap is below 3.5 eV. As expected, an additional doped layer slightly reduces the value of *E_g_* [35]. The index of refraction is influenced by deposition temperature. Samples deposited at lower temperatures (S-590 and B-590) have a higher index of refraction. In addition, comparing samples deposited at the same temperature, the samples with higher surface roughness have lower index of refraction. According to the effective medium approximation [36], the surface layer of a thin film with a rough surface can be considered a mixture of bulk material and air. The effective density and index of refraction of such a layer are lower than those of the bulk material.

At the interface formed by two different optical media with a roughness *σ*, the light is scattered into four different components: specular reflection (*R_s_*), specular transmittance (*T_s_*), diffuse reflection (*R_d_*), and diffuse transmittance (*T_d_*) [13]. The ratio of directly and diffusely transmitted light can be quantitatively expressed by the haze ratio.

**Figure 9:**
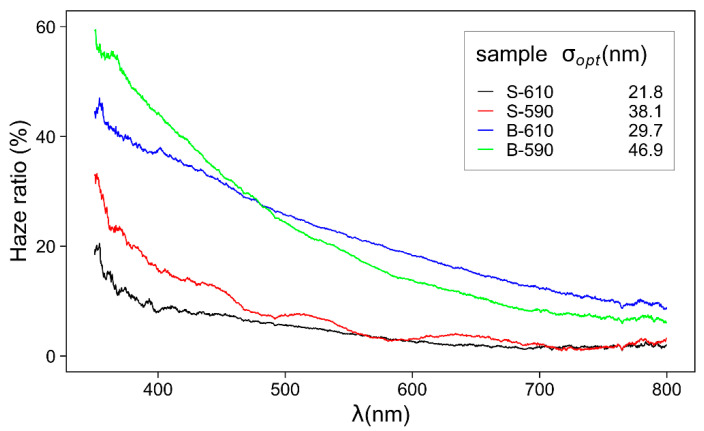
Spectral distribution of transmittance haze ratio for single- and double-layer SnO_2_ thin films. Surface roughness values are obtained by Equation (6).

After measuring the specular and total transmittance, the transmittance haze ratio can be calculated as:

(5) $$Haze\left[\%\right]=\frac{T_{d}}{T_{tot}}100\%$$

where *T_d_* is the diffusely scattered transmittance, *T_tot_* is the total transmittance *T_tot_* = *T_d_* + *T_s_*. The spectral distribution of the haze ratio is presented in Figure 9. The diffusely scattered component is more pronounced for the shorter wavelength range. That is in accordance with the theoretical model where the diffuse transmittance (*T_d_*) can be expressed as [13,37]:

(6) $$T_{d}=T_{s}\left[1-e^{-{\left(\frac{4\pi \left(n_{air}-n_{film}\right)\sigma_{opt}}{\lambda }\right)}^{2}}\right]$$

where T_s_ is the specular transmittance of a perfectly smooth surface, n_air_ and n_film_ are the index of refraction of air and TCO thin film, *σ_opt_* is the surface roughness, and λ is the light wavelength. The surface roughness (*σ_opt_*) is calculated using Equation (6) and compared to the results obtained by AFM (Table 2). *σ_opt_* and *σ_q_* are similar and proportional to the domain sizes, thus confirming the estimation procedure. According to Equation (6), the samples with larger roughness have larger diffuse reflectivity and hence lower transmittance especially in UV range.

## 4. Discussion

We have applied several different analytical techniques for the structural and surface morphology analysis of APCVD deposited SnO_2_ thin films. The applied techniques vary by the used probe (electrons, mechanical probe, X-rays, UV-Vis-NIR light), the size of the analysed sample area ranging from 1 μm^2^ to 100 mm^2^, and the resolution ranging from 1 nm to 1 μm (Table 4). The requirements and the complexity of the sample preparation procedure should not be neglected. Some of the applied techniques provide the same information but with different scales and resolutions.

By combining the results obtained by all applied analytical methods we can obtain a precise description of the SnO_2_ thin film’s surface morphology. The samples have mostly a homogenous surface over a large area except for rare defects (cracks and deep holes). The layer structure and surface morphology are very sensitive to the sample preparation parameters (temperature, thickness, precursor mixture content). The surface is dominated by randomly oriented pyramid-like shapes which correspond to grain corners with large flat inclined sides.

According to AFM and TEM images, samples deposited at lower temperatures have a significantly higher roughness, which results in more efficient light scattering, leading to a higher haze ratio and a longer optical path through the film. Also, bilayer films show an increment of both roughness and haze ratio, compared to single layers. This extends down to the nanometre scale, as skewed scattering (40° to 70° range) in GISAXS. This is confirmed through similar fractal dimensions obtained from GISAXS and AFM, with a ~1–10 nm and ~10–1000 nm probed size range, respectively. Enhanced diffuse light scattering is very important for efficient light absorption in the active part of thin film solar cells and an improvement of the solar cell’s conversion efficiency.

As we presented in the manuscript, many of the parameters that describe the surface morphology can be estimated by two or more applied analytical techniques (for example surface roughness and fractal dimensions). All of them are qualitatively in agreement (variation from sample to sample, trend related to deposition parameters). Quantitative variations are related to specific properties of each analytical method: resolution, the scale at which information is provided, and the scanned sample area/volume.

TEM cross-section images provide the surface profile, film thickness, and roughness but only at one line (cut) several micrometres long. In addition, SAED provides information about the crystallinity and nanocrystal orientation. However, the sample preparation for TEM is complicated and time consuming and includes ion etching. Furthermore, the influence of the etching process on the sample surface morphology and crystallinity cannot be completely neglected.

Compared to TEM, AFM provides the 2D topography of a sample surface for a wider area (10 μm^2^). It is very sensitive in the direction perpendicular to the sample surface. However, it does not give information about the sample thickness. The finite size of the scanning tip restricts the resolution and detection of sharp edges. It is not destructive and does not require a special sample preparation procedure.

GISAXS is a technique that provides information about the structure and surface morphology in reciprocal space, and for quantitative data analysis, it is necessary to assume an appropriate theoretical model to transform the measured information about structure and morphology into direct space. Compared to AFM and TEM it provides information statistically averaged over a large sample area.

## 5. Conclusions

We have examined the structure and surface morphology of SnO_2_ thin film samples deposited by APCVD on soda-lime glass. Such films are widely used as front transparent electrodes in thin-film solar cells. The characterisation was conducted by TEM, AFM, and GISAXS. We have compared the results obtained by each technique and discussed the differences and pros and cons of each experimental technique. The used techniques provide information about the sample surface morphology at different scales in the range from 1 nm to 1 μm, yet with different statistical significance, and averaged over different sizes/areas of the sample. Some of them are not destructive and can be easily applied without previous sample preparation (AFM, GISAXS), while others require special and time-consuming sample preparation such as TEM. Some of them provide direct insight into the sample structure and surface morphology but are limited to a very small area of the sample (AFM, TEM), while others provide information statistically averaged over a large area of the sample. The data are provided in reciprocal space and require the application of a proper theoretical model to obtain real space quantitative information (GISAXS). By combining the information obtained from all applied characterisation techniques, we were able to provide a detailed structural and surface morphology description of the prepared SnO_2_ thin film samples.

In addition, we showed that the surface morphology (surface roughness) has a significant influence on the optical properties (diffuse transmittance, haze ratio) that condition light-harvesting through the obtained thin films and should be taken into consideration during light harvesting analysis.

## Figures and Tables

**Table 1:** SnO_2_ layer deposition parameters (doping, deposition temperature), thickness estimated from optical measurement (UV-Vis-NIR transmittance), and TEM.

Sample Name	Sample Type	Deposition Temperature (°C)	Thickness UV-Vis-NIR (nm)	Thickness TEM (nm)
S-610	undoped single layer	610	300 ± 22	308 ± 18
S-590	undoped single layer	590	390 ± 38	270 ± 40
B-610	undoped/doped bilayer	610	710 ± 30	582 ± 51
B-590	undoped/doped bilayer	590	920 ± 47	811 ± 36

**Table 2:** Result of AFM images analysis: surface roughness parameters σ_q_ and σ_a_, z range *Z_max_−Z_min_*, fractal dimension calculated by variance method, cube counting method. and triangulation method. Results are obtained by Gwyddion software [18].

Sample	Surface Roughness		*Z_max_*−*Z_min_* [nm]	Fractal Dimension		
	*σ_q_* [nm]	*σ_a_* [nm]		Variance	Cube Counting	Triangulation
S-610	19.3	13.7	319	2.58	2.29	2.37
S-590	40.2	28.4	444	2.65	2.37	2.47
B-610	29.2	22.0	458	2.67	2.43	2.54
B-590	43.9	34.2	414	2.61	2.37	2.49

**Table 3:** Values of the optical constants of SnO_2_ thin films: optical band gap (*E_g_*), effective index of refraction (*n_eff_*) and surface roughness, determined from the haze ratio (*σ_opt_*) determined from UV-Vis-NIR experiment.

Sample Name	*E_g_* (eV)	*n_eff_*	*σ_opt_* (nm)
S-610	3.4 ± 0.1	2.5 ± 0.1	21.8
S-590	3.5 ± 0.1	2.9± 0.1	38.1
B-610	3.3 ± 0.1	2.2± 0.1	29.7
B-590	3.3± 0.1	2.4± 0.1	46.9

**Table 4:** Characteristics summary of all used experimental techniques for surface morphology analysis of SnO_2_ thin films on glass substrate.

Experimental Technique	Information Provided by Analysis	Size of Sample Area Analysed	Resolution	Pros	Cons
TEM	thickness, roughness, crystallinity, surface profile	Only 1D line 1–10 μm	1 nm	In-depth profile of sample	Pure statistics, complicated sample preparation
AFM	Only top surface morphology, roughness, fractal dimension	1–10 μm squared area	10 nm	Image of large area sample surface	Finite tip size
GISAXS	Shapes at the surface, fractal dimensions	5 × 50 mm	1 nm	Good statistics over large area of sample	Information in reciprocal space
TR	Roughness, thickness	10 × 10 mm	100–1000 nm	Simple and not expensive technique	
